# Effects of Recycled Polymer on Melt Viscosity and Crystallization Temperature of Polyester Elastomer Blends

**DOI:** 10.3390/ma16176067

**Published:** 2023-09-04

**Authors:** Ji-Eun Lee, Jin-Woo Lee, Jae-Wang Ko, Kyung-Il Jo, Hyun-Ju Park, Ildoo Chung

**Affiliations:** 1 Korea Institute of Footwear & Leather Technology, 152 Dangamseo-ro, Busanjin-gu, Busan 47154, Republic of Korea; jelee@kiflt.re.kr (J.-E.L.); jwko@kiflt.re.kr (J.-W.K.); kijo@kiflt.re.kr (K.-I.J.); hjpark@kiflt.re.kr (H.-J.P.); 2 Department of Polymer Science and Engineering, Pusan National University, Busan 46241, Republic of Korea; 3 Department of Organic Material Science and Engineering, Pusan National University, Busan 46241, Republic of Korea

**Keywords:** recycled polymer, thermoplastic polyether ester elastomer, circular economy, polyester elastomer, melt viscosity, crystallinity

## Abstract

As the world is paying attention to the seriousness of environmental pollution, the need for a resource circulation economy is emerging due to the development of eco-friendly industrial groups. In particular, the recycling of thermoplastic elastomers without cross-link has been highlighted in the plastics field, which has rapidly developed the industry. Growing interests have been directed towards the advancement of thermoplastic polyether–ester elastomer (TPEE) as a material suitable for the circular economy owing to its remarkable recyclability, both in terms of mechanical and chemical processes. Due to its excellent processability, simple mechanical recycling is easy, which is a driving force towards achieving price competitiveness in the process. In molding TPEE resin, it is essential to check the thermal properties of the resin itself because the thermal properties, including the melting and crystallization temperatures of the resin, depend on the design of the polymer. In this study, the thermal and mechanical performances of TPEE blends were evaluated by manufacturing compounds by changing the amount of recycled resin and additives. When the recycled resin was added, the melt flow index (MFI) changed rapidly as the temperature of the melt flow index measurement increased. Rapid changes in MFI make the fiber spinning process uncontrollable and must be controlled by optimizing the addition of compatibilizers. Based on the thermal property results, compatibilizers such as Lotader and Elvaloy series exhibited minimal change in glass transition temperature, even with greater amounts added. This makes them well-suited as compatibilizers for fiber spinning.

## 1. Introduction

Thermoplastic polyether–ester elastomer (TPEE), with both the mechanical strength (hardness) of plastic and the flexible properties (elasticity) of rubber, is a block copolymer composed of rigid poly (butylene terephthalate) (PBT) segments serving as hard segments and soft segments consisting of aliphatic ether ester. It demonstrates excellent durability, heat resistance, abrasion resistance, impact resistance, and chemical resistance, with a lifespan three times longer than that of general rubber, and it also is twice as light as general rubber [1,2,3,4,5,6]. Since its hard segment (with excellent mechanical strength) and its soft segment (with a rubber-like flexibility) are combined with each other, TPEE is a high-functional material that combines the elasticity of rubber and the molding processability of plastic. Due to its low specific gravity, it serves as a substitute for vulcanized rubber and polyvinyl chloride (PVC), and it can be used in a wide range of applications such as those pertaining to the automotive industry, household appliances, construction materials, and clothing, with a continuous growth of demand.

Among them, TPEE is mainly used in areas where shock absorption, impact resistance, flex resistance, sealing, elasticity, oil resistance, chemical resistance, and sufficient strength are required. It is made up of at least two distinct domains that are a mix of crystalline hard and amorphous soft phases. A reversible phase is formed due to the molecular motion in a rubbery state, and elastomeric property is obtained by the flexible soft segment with amorphous areas at high temperatures. Hydrogen bonding, polar interactions, and crystallization in the hard domain, which melts at high temperatures, form physical crosslinks. It can be used for a variety of purposes, such as for reinforcing wear-resistant high- and low-temperature properties in the field of automotive parts, high- and low-temperature-resistant wire sheaths, hydraulic hoses, shoe materials, transmission belts, flexible couplings, silencer gears, elevator slides, chemical equipment pipeline valve corrosion protection, and so on [7,8,9,10,11,12,13,14].

Due to worldwide interest in the seriousness of environmental pollution and the development of eco-friendly industries, the easy recycling and eco-friendliness of non-crosslinked thermoplastic elastomers are highlighted in light of the need for a circular economy. A resource circulation economy does not refer to a linear material flow of ‘consumption–disposal’ but refers to an economic system in which materials input to the economy are not discarded but are instead repeatedly used as useful resources within the economy. As a material that can be applied to such a circular economy, TPEE, with excellent recyclability, is attracting attention. Increasing the R&D activities of numerous companies involved in the production, distribution, and recycling of TPEE compounds is expected to spur investment in the market and propel growth. TPEE not only has good performance; it is also easy to recycle as a type of PET; waste and final waste from the production process can be directly returned for reuse. It is easy to recycle mechanically due to its excellent processability, which is the driving force of price competitiveness in the process [15,16,17,18,19,20,21,22].

On the other hand, TPEE can withstand a wide temperature range. It can maintain its flexible properties even at low temperatures and resist deformation at high temperatures. Therefore, it is essential to confirm the thermal properties of the resin itself, such as the melting temperature and crystallization temperature of the resin, for accurate polymer design of TPEE with a wide temperature range. The cylinder temperature is basically set low for soft resin and high for hard resin. Mold shrinkage is also affected by the structure of the polymer used in injection molding, such as type, composition ratio, molecular weight distribution of hard/soft segments, molecular orientation, and crystallization [23,24,25,26,27,28]. Consequently, it is influenced by the thickness of the molded product, with thinner products experiencing a lower shrinkage rate.

Thermoplastic polymers such as TPEE can be easily recycled due to their thermoplastic properties but have the disadvantage of deteriorating physical properties depending on the recycling method and number of cycles. Both virgin and recycled TPEE are long-lasting and possess high-impact strength, excellent fatigue, as well as tear and abrasion resistance along with good electrical properties. Depending on the type of TPEE and the desired application, the material properties can be further improved using additives such as flame retardants, UV stabilizers, antistatic and foaming agents, or monomer resins for rheology control. Among the many ways to control the structure and final properties of TPEE, a convenient method is to blend it with polymers with a variety of unique properties including crystalline and amorphous polyesters and polyolefins. Some new TPEE-based blend materials and/or alloys with excellent flexural fatigue properties and extended service temperature ranges have been successfully developed using blend technology [29,30,31,32].

In this study, a solvent-free thermoplastic polyester elastomer composition that does not use a solvent was prepared and evaluated. In addition, various compatibilizers were added to the prepared compound to control the chemical properties of the compound, and the meltability and crystallinity of the compound were adjusted to improve the moldability and mechanical properties of the polymer.

## 2. Materials and Methods

### 2.1. Materials

TPEE was obtained from LG chemical Co. Ltd. (Seoul, Republic of Korea) with different hardness levels of Keyflex BT1172D and BT1155D. Recycled TPEE (R-TPEE) was used after receiving waste resin obtained from the production process of textile products with the same hardness level and grade (Jeongsan International, Busan, Republic of Korea).

The radical initiator applied in the reaction extrusion process was Perkadox BC-FF of the Dicumyl peroxide (DCP) series (Nouryon, Amsterdam, The Netherlands). The following four polymer compatibilizers were used to enhance physical properties by modifying the resin: Fusabond N525, Elvaloy 3427AC (DuPont, Wilmington, DE, USA), Lotader AX8900, and Lotryl30BA02 (Arkema, Paris, France). Fusabond is an ethylene copolymer modified with anhydride, whereas Elvaloy is a copolymer of ethylene and butyl acrylate containing 27% butyl acrylate and can be used for coextrusion and compounding. For Arkema grades, Lotader is a random terpolymer composed of ethylene methyl acrylate-glycidyl methacrylate, while Lotryl is a random copolymer of ethylene and butyl acrylate produced by high-pressure radical polymerization process.

### 2.2. Manufacturing Process of Specimens

The blends of TPEE from waste resin generated during the production process and the existing high hardness resin were prepared with various mixing ratios and evaluated to compare their thermal and physical properties. Both pre-dried TPEE resin and waste at 90 °C for 24 h before use were extruded at 230 °C, and their physical properties were evaluated.

The TPEE blends were melt-blended using a corotating-type twin-screw extruder (EM Korea, Changwon-si, Republic of Korea, L/D = 40, 19 Φ) at a screw speed of 150 rpm. The temperature from the feeder to the die was set to 180–240 °C, while maintaining a discharge rate of 10 kg/h. The extrusion process proceeded through a conveying zone, a kneading zone, a reverse zone, a conveying section for pressure generation. After cooling in a cooling water bath, the blends were obtained in the form of pellets through a pelletizer and dried under vacuum at 60 °C for 24 h to constant weight. The tensile specimens were fabricated by changing the ratios of TPEE resin and waste at an injection temperature of 230 °C using injection (PR170HY, 150 kgf/cm^2^ clamping force, Dongshin Hydraulics, Busan, Republic of Korea) in accordance with ASTM standard (ASTM D 638) [33].

### 2.3. Evaluation Methods

The mechanical properties of TPEE composites were measured using a universal testing machine (UTM) (Instron M4465, Instron, Norwood, MA, USA) at a cross-head speed of 200 mm/min and a cell load of 5 kN. The glass transition temperature ($T_{g}$) and melting temperature ($T_{m}$) of TPEE composites were measured under nitrogen flow using a differential scanning calorimeter (DSC, TA instruments Q 100, TA Instruments, New Castle, DE, USA) at a heating rate of 10 °C/min. The measurement temperature was measured in 2 cycles at a rate of 10 °C/min from 0 °C to 250 °C. Pyrolysis initiation temperature were determined by thermogravimetric analysis (TGA; Q500, TA instruments, New Castle, DE, USA) at a heating rate of 10 °C/min under nitrogen flow.

MFI is a measure of the ease of flow of a thermoplastic polymer melt according to ASTM D1238 [34]. The MFI measurement conditions of the TPEE used in this study were measured under the conditions of an available load of 2.16 kg, an available temperature from 200 to 240 °C, and a shear rate in the range of 30 to 1000 s^−1^. The MFI test was measured repeatedly until the same trend appeared at least three times.

The tensile strength test of the injection specimen was carried out according to the ASTM standard (ASTM D 638 [33]). Injection pressure and holding pressure were set to 1625 bar, and injection and holding pressure were maintained for 3 s and 5 s, respectively. After injection, the mold was demolded with a cooling time of 30 s. Specimens used for measurement were prepared in the form of dumbbell No. 3. The tensile test was measured with a UTM at room temperature and a speed of 500 mm/min.

The tensile strength test was performed by measuring at least 5 specimens and obtaining the average. Hardness was determined with Durometer Shore D type based on ASTM D2240 [35] (GS-706N, GS-702N, Teclock, Japan).

## 3. Results and Discussion

### 3.1. Measurement of Basic Properties for Fiber Spinning

#### 3.1.1. Physical Characterization of TPEE Compositions

**Figure 1:**
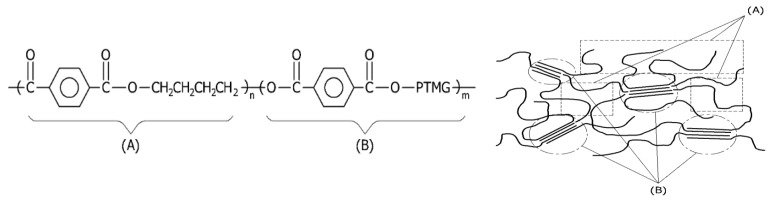
General structural schematic diagram of polyether–ester block copolymer: (A) hard segment; (B) soft segment.

**Figure 2:**
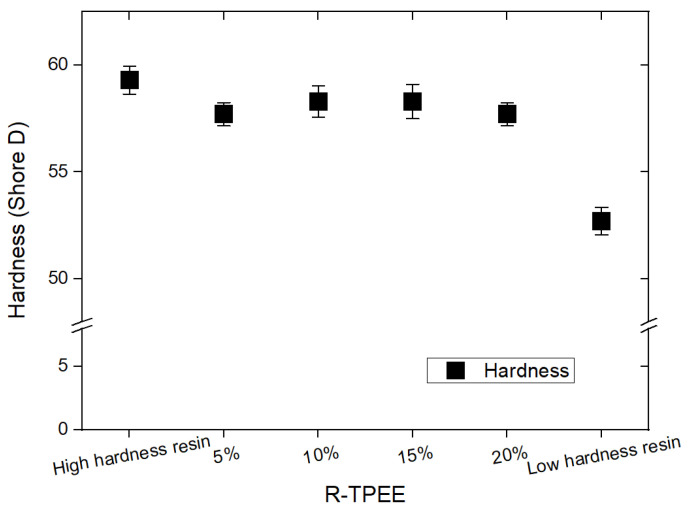
Hardness of thermoplastic polyether–ester elastomer compositions.

To analyze the properties of TPEE composites, it is necessary to understand the structure of polyether ester block copolymer, which is a polymer that can be divided into soft and hard segments, as shown in Figure 1. It is possible to design a polymer with desired physical properties by controlling the contents of hard and soft segments. The hardness of recycled TPEE (R-TPEE) prepared from waste TPEE was analyzed using a Shore D type hardness tester and expressed as the average values from at least three or more samples. As shown in Figure 2, the hardness values ranged from 52.7 to 59.3, respectively. Tensile strength and hardness were measured using resins with relatively different hardness. Test pieces using recycled resin were measured for tensile strength and hardness by adding 5 to 20% of R-TPEE in a weight ratio to the base polymer of the high-hardness resin. Overall, when adding high-hardness recycled resin, it was found that the hardness was lower than that of the test piece manufactured using the first high hardness resin, but it was confirmed that the hardness was higher than that of the low hardness resin. The MFI indicates the weight extruded for 10 min at a constant temperature and a prescribed force. In this study, all the resin flowed in less than 10 min, even under the prescribed force in the test specimen made by adding 20 wt.% or more of recycled resin. Therefore, in this test piece, the numerical value was impossible to measure by the method of measuring the MFI. In addition, it was impossible to manufacture a test piece with a high MFI even in the production of a test piece using injection. It was found that the hardness decreased as the content of R-TPEE resin increased, although the rate of decrease in hardness was judged to be insufficient as the content of the resin increases.

**Figure 3:**
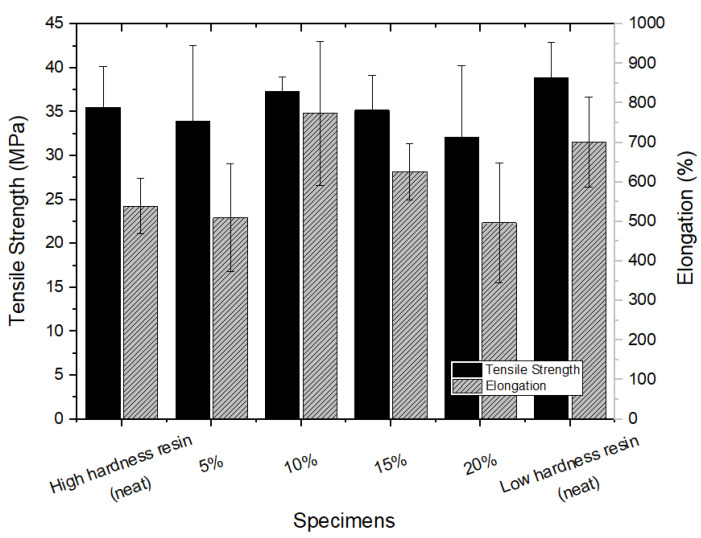
Tensile strength and elongation at break of recycled thermoplastic polyether–ester elastomer mixed resin.

All the test specimens with R-TPEE added from 5% to 20% showed a slight change in hardness within 5% compared to the high-hardness resin test specimen in the neat state presented in Figure 2. The R-TPEE composite resin was formulated using waste TPEE resin, and its tensile strength and elongation were evaluated using UTM equipment by varying the recycled resin mixing ratio and different hardness. Three samples of each formulation were used to find their average values with standard deviations, and they are presented in Figure 3.

#### 3.1.2. Thermal Properties of TPEE Compositions

**Figure 4:**
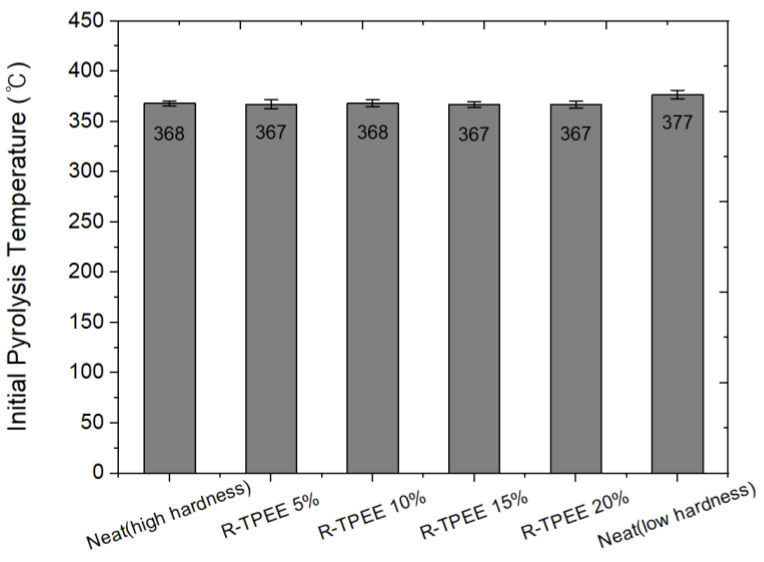
Thermal decomposition initiating temperature of the composition by the blend ratio of recycled thermoplastic polyether–ester elastomer mixed resin.

The thermal properties of TPEE were confirmed through TGA analysis. The initial pyrolysis temperature of low-hardness TPEE shown in Figure 4 was about 10 °C higher than that of high-hardness TPEE. Furthermore, the blend with recycled TPEE showed no differences in its initial pyrolysis temperatures, regardless of the contents. In the case of char (pyrolyzed carbonaceous material), there was almost no effect of hardness and recycled TPEE content at about 3–4% level. Based on the above comprehensive characterization of TGA analyses, it can be confirmed that the addition of recycled TPEE chips does not affect the thermal decomposition characteristics of the resin itself.

**Figure 5:**
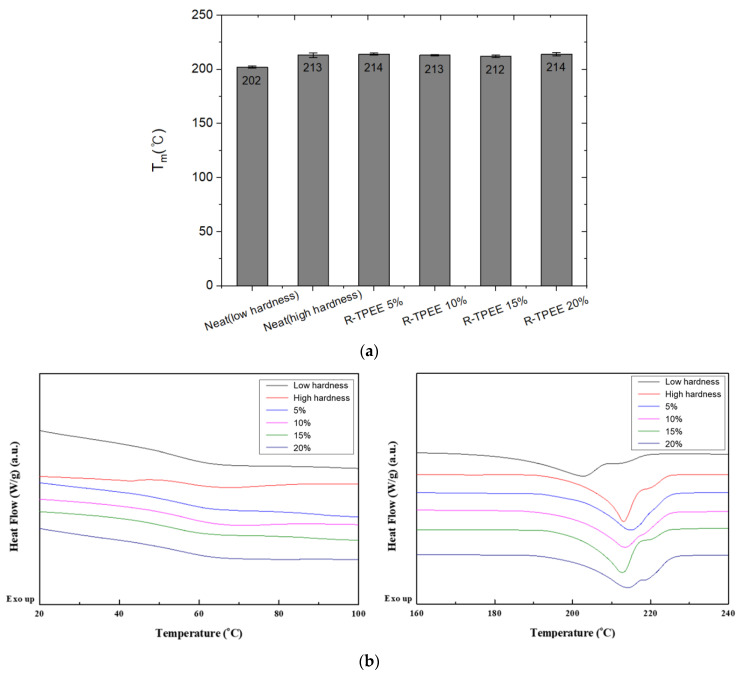
Comparison of thermal properties as a function of the blend ratio of recycled thermoplastic polyether–ester elastomer blended resins: (**a**) T_m_; (**b**) DSC graph.

DSC analysis was performed to confirm the $T_{m}$ and $T_{c}$ of TPEE composition. The temperature was measured in two cycles at a rate of 10 °C/min from 0 °C to 250 °C. As shown in Figure 5a, the $T_{m}$s of the low-hardness and high-hardness resins have a difference of about 10 °C, and they do not change with varying the amount of recycled TPEE, meaning that the addition of recycled TPEE resin did not affect the thermal properties of the blends. 

**Figure 6:**
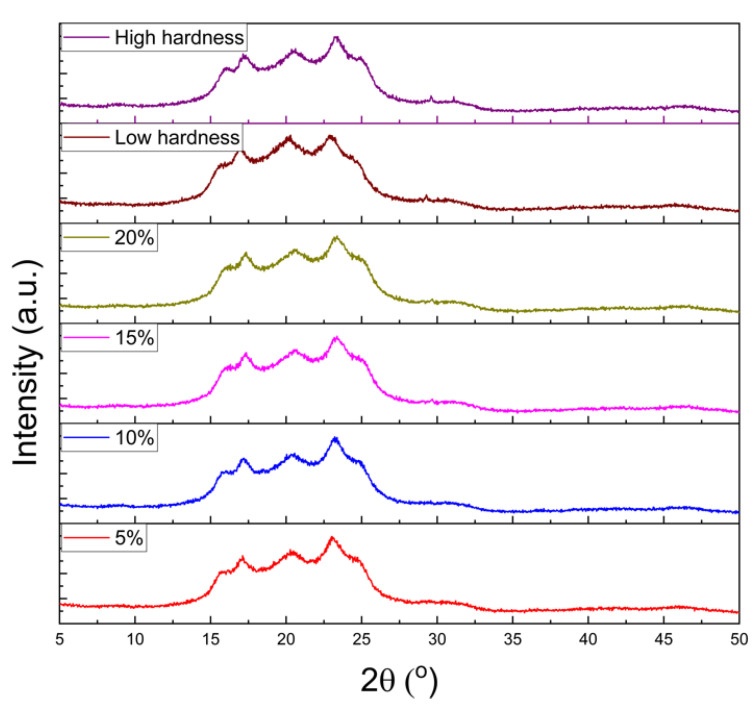
XRD analysis results by recycled thermoplastic polyether–ester elastomer content.

WAXD analysis was performed to confirm structural characteristics as a function of the blend ratio of R-TPEE mixed resin at a wavelength λ of 0.154 nm and presented in Figure 6. TPEE showed a very weak diffraction peak at about 2θ = 15.9° regardless of its hardness. Additional diffraction peaks were observed at 2θ of 16.4°, 17.9°, 21.3°, and 22.5°, which were arisen from the diffraction peaks of the PBT segment responsible for crystallinity in TPEE. They were clearly seen when high hardness TPEE was added due to the high content of crystalline PBT. Significant shifts or intensity changes were not observed, even when recycled chips were mixed, indicating that the original structure of the material remained unchanged even after recycling.

**Figure 7:**
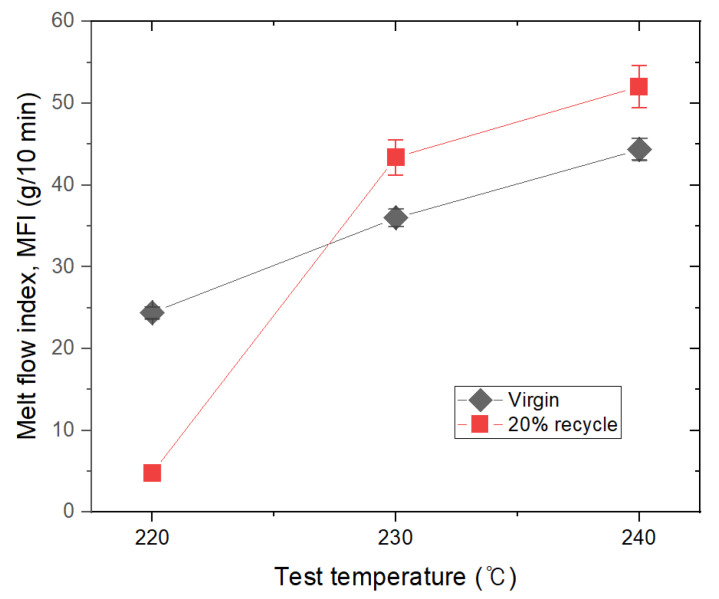
MFI value changes as a function of temperature of composition containing 20% recycled thermoplastic polyether–ester elastomer.

As a result of confirming the basic characteristics of the composition by controlling the content of recycled TPEE, it was confirmed that the physical properties of the basic resin did not change significantly, even when the maximum amount of 20% was used in this study. Various physical property evaluations were performed in order to utilize the composition prepared through this result in fields such as fiber spinning. Figure 7 is a graph showing changes in the MFI value according to the temperature of the TPEE composite composition containing 20% of R-TPEE. As the melting temperature increased, the viscosity increased more in the test piece to which 20% of R-TPEE was added than in the test piece to which nothing was added. This might have increased the MFI value due to a decrease in the molecular weight of the added R-TPEE. If the MFI value is high, the flowability occurs excessively during product molding, resulting in poor workability.

### 3.2. Characteristic Evaluation by Addition of Compatibilizer

A compatibilizer was added to compensate for the substantial reduction in melt viscosity caused by the incorporation of recycled resin (R-TPEE). As depicted in Table 1, four types of compatibilizers, along with a radical initiator, were introduced into the blended compositions of TPEE and R-TPEE through a reaction extrusion process and then used to evaluate their properties.

**Figure 8:**
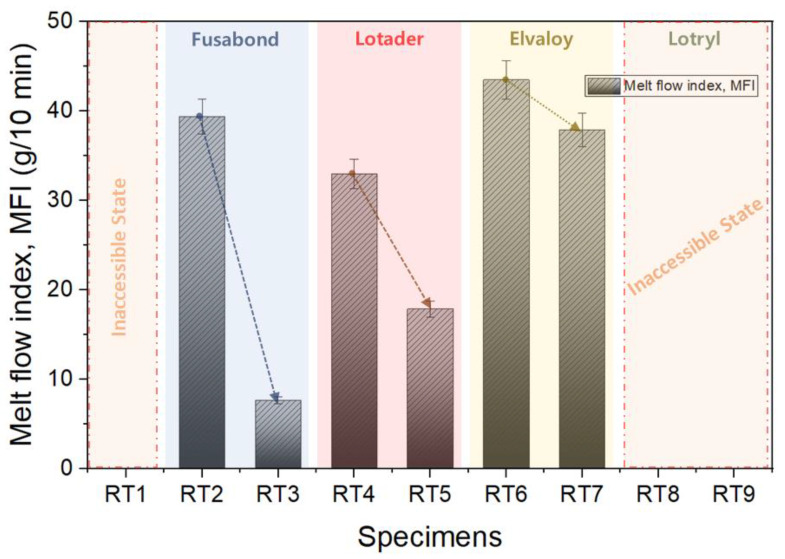
Effect of the type of compatibilizer on MFI.

Figure 8 shows the consequent changes in the melt index resulting from the addition of a compatibilizer. Existing TPEE had a significantly lower viscosity when melted. Thus, it was not measured at a level that flowed like water. TPEE without compatibilizer was not suitable for use in a fiber spinning process. Among these, the melt viscosities of the blended compositions containing three compatibilizers, namely, as Fusabond N525, Lotader AX8900, and Elvaloy 3427AC, increased with the increasing amount of compatibilizer. However, melt flow indices of RT1, RT8, and RT9 samples could not be measured because their viscosities during melting were significantly lower, like that in a conventional TPEE. The MFI of the specimen mixed with Fusabond-type compatibilizer decreased the most rapidly with the increase in compatibilizer. In the case of the Elvaloy-based formulation composition, it was confirmed that the change in MFI was the smallest as a function of the change in the additional amount of compatibilizer. For the blended compositions with Fusabond and Lotader, when the amount of addition increased from 10% to 20%, the melt index decreased rapidly. Therefore, if the amount of compatibilizer increased by more than 20%, it is judged that the molding of the product was difficult due to the high flowability of the resin. Since Elvaloy did not induce a reduction in the melt index of the blends, even when utilized at elevated compatibilizer levels, it could be used with a higher ratio of compatibilizer. Because the melt index for fiber spinning generally ranges from 30 to 50 g/10 min, the blends of RT2, RT4, RT6, and RT7 show promise as potential candidates for fiber spinning based on their melt index values. For the blended compositions with the Elvaloy series, it was determined that those with higher content would not cause any issues with the melt index for spinning. However, it would be necessary to conduct careful examinations of the changes in physical properties depending on the content of the compatibilizer.

**Figure 9:**
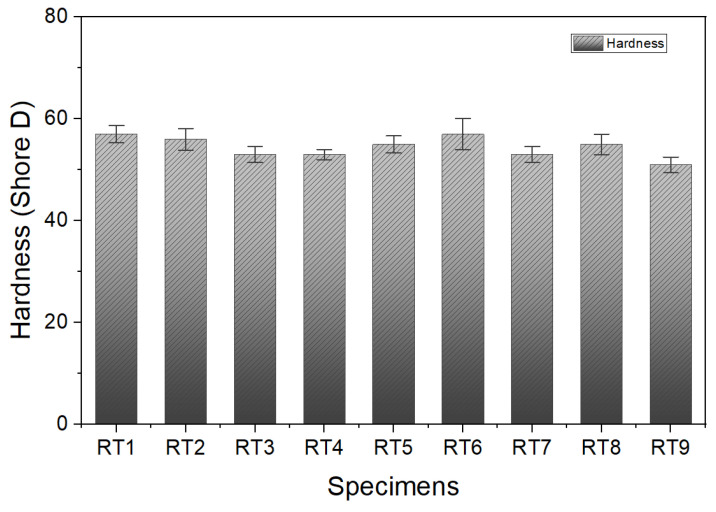
Effect of the type of compatibilizer on hardness.

Figure 9 shows the results of hardness measurements conducted to confirm the change in physical properties as a function of the use of compatibilizer. In the case of Lotader AX890, the hardness increased with the increase in compatibilizer. On the other hand, for the remaining specimens, a tendency to decrease in hardness was confirmed as the ratio increased. Most specimens did not show any significant changes in hardness, meaning that the addition of compatibilizer at the level performed in this study did not significantly affect the hardness.

**Figure 10:**
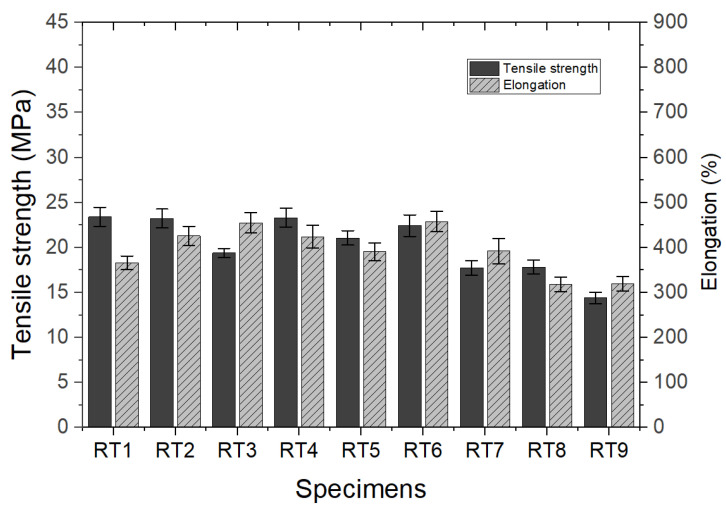
Tensile strength and elongation at break by compatibilizer type and compounding ratio.

Mechanical properties such as tensile strength and elongation at break were also evaluated according to the type and mixing ratio of the compatibilizer and are presented in Figure 10. The RT4 sample showed the most similar tensile strength to that of the specimen without additives, whereas its elongation was slightly improved. Compared to that of RT1, in the case of blended compositions with Elvaloy such as RT6, tensile strength decreased with the increasing content of compatibilizer, with a slight improvement of elongation. Consequently, these compositions would be potential candidates for the fiber spinning process.

**Figure 11:**
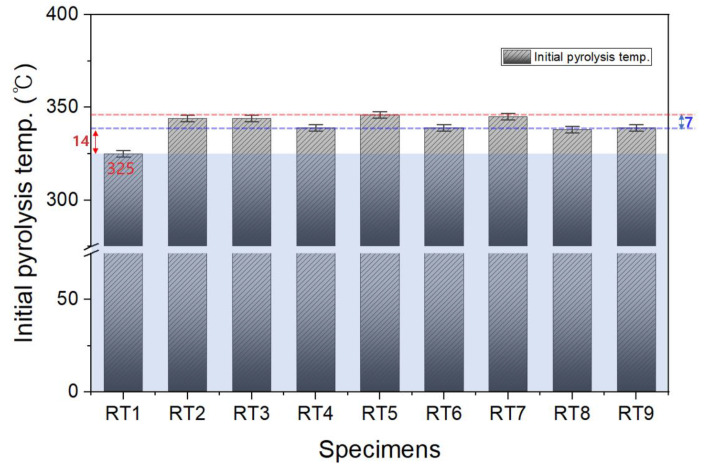
Thermal properties depending on the compatibilizer type and mixing ratio.

**Figure 12:**
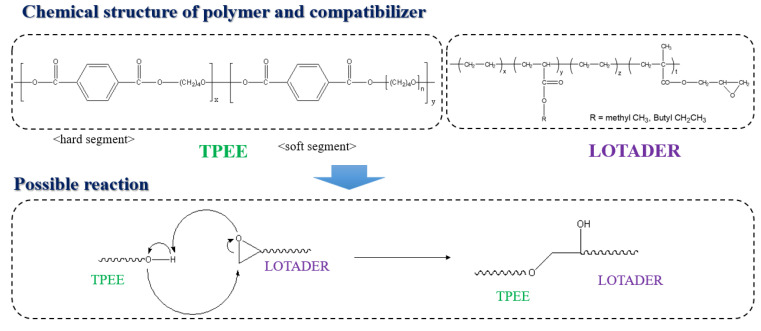
Possible reaction between TPEE and Lotader during melt blending.

TGA and DSC analyses were performed for thermal characterization. Results for the initial pyrolysis temperature are shown in Figure 11. In the graph, the blue line is the minimum value of the initial pyrolysis temperature when the compatibilizer is added, and the red line is the maximum value. It was confirmed that the thermal decomposition temperature increased due to the stronger bonding between polymers and the compatibilizer. The initial pyrolysis temperatures did not show significant changes depending on type of compatibilizer, with a temperature difference of 7 °C between the highest and lowest. Figure 12 illustrates a potential reaction between TPEE and Lotader during melt-blending. This suggests that the glycidyl methacrylate group of Lotader and the hydroxyl group of TPEE could react to increase their molecular weight. This reaction could contribute to the control of MFI and the increase in heat resistance, leading to the increase in thermal decomposition temperature due to the strong bonding between polymers and compatibilizers.

As one of the simple methods for measuring the molecular weight and molecular weight distribution of polyolefin-based materials, the S.Ex (stress exponent) method was used to determine the molecular weight distribution after measuring the melt index based on the correlation between the melt index and molecular weight distribution. The flowability index varies depending on the molecular weight distribution, type of polymer, and molecular shape, but in general, it is simply used as an index showing correlation with molecular weight in polymers. In this study, since the molecular weight distribution could not be confirmed due to the impossible conditions of GPC measurement, the flowability index was measured and used as a factor to confirm the correlation with the molecular weight. MFI is measured by applying a constant load to the molten resin and measuring the amount of resin that passes through the nozzle at a certain time. The melt index does not actually mean the molecular weight; it is a method that can compare molecular weight by comparing the melt index values of polymer resins [36,37]. Lee et al. also conducted a study to determine the molecular weight and molecular weight distribution of thermoplastic polymer materials using rheological properties. The standard of the strain value as a function of the MFI of the sample was selected, and the result was derived for the molecular weight distribution [38]. In general, the relationship between molecular weight and the melt index is inversely proportional, and materials having a high molecular weight (low melt index) improve physical properties such as stiffness, stress uniformity, chemical resistance, and elongation, but processability decreases due to a decrease in viscosity.

**Figure 13:**
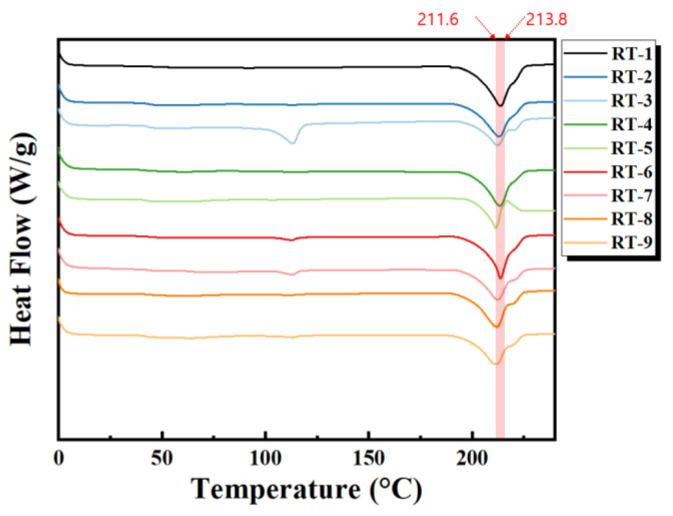
Melting point by the compatibilizer type and mixing ratio based on DSC analysis.

Therefore, the increase in thermal decomposition temperature and the changes in viscosity and mechanical strength were determined for the following reasons. Usually, as the size of the block of the hard segment increases due to the increase in the molecular weight of soft segment (poly(tetramethylene ether)glycol, PTMG), presented in Figure 12, both strength and heat resistance are increased. However, looking at the results in Figure 8 and Figure 10, the tensile strength was not improved, despite the decrease in MFI, due to the increase in molecular weight. These results indicate that Lotader compatibilizer could selectively react with the soft segment of TPEE, not with the hard segment, leading to an increase in the molecular weight of the soft region. Figure 13 displays the DSC thermogram confirming the melting temperatures of the blends based on the type and mixing ratio of the compatibilizer. It was observed that all specimens exhibited similar melting temperatures, suggesting that the addition of the compatibilizer did not significantly affect the melting temperature. Because the melting temperatures of Fusabond N525 and Elvaloy 3427AC are 58 °C and 94 °C, respectively, the melting peak around 112 °C can be attributed to either a reactant generated through a side reaction with TPEE or a crosslinking agent.

**Figure 14:**
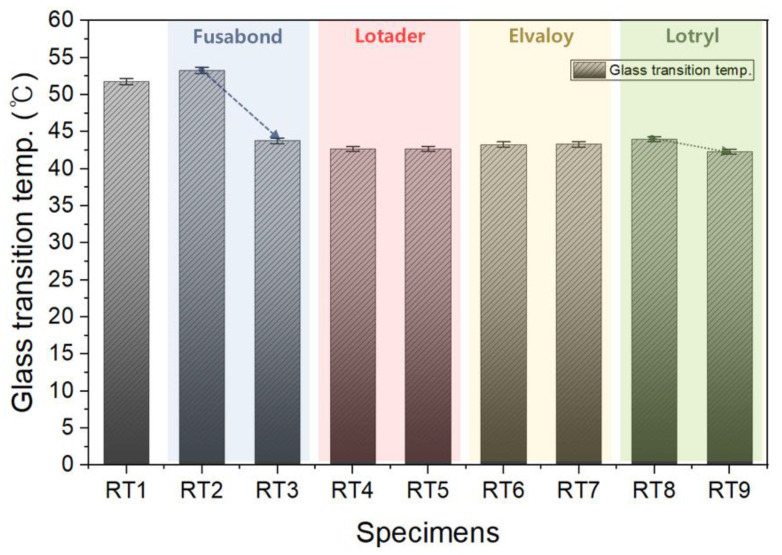
Glass transition temperature depending on the type of compatibilizer and the mixing ratio.

Figure 14 shows the glass transition temperature depending on the type and amount of compatibilizer added. The glass transition temperature is an important variable that determines the cooling method and the rate for fiber spinning after melting in the fiber spinning process. If the glass transition temperature shows a sharp change depending on the amount of the compatibilizer added, it is difficult to finely control the content, resulting in great difficulty in manufacturing. In the case of the specimen containing the Fusabond-based compatibilizer, the glass transition temperature changed significantly by more than 10 °C as the amount of addition increased. On the other hand, insignificant changes of less than 1.5 °C were observed in other compatibilizers. Therefore, the Fusabond-based compatibilizer was determined to be unsuitable as an additive for mass production of fiber spinning; it may have a large variation in glass transition temperature depending on the amount added. The glass transition temperature of the compatibilizers of the Lotader series and the Evaloy series did not change, even with an increase in the amount added. Therefore, it was considered that these two series of compatibilizers were suitable as compatibilizers for fiber spinning.

**Figure 15:**
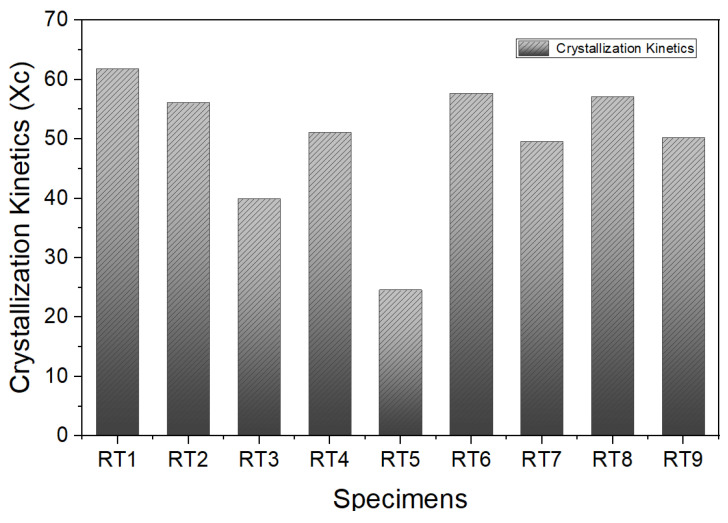
Crystallization level by the difference in compatibilizer type and mixing ratio.

Figure 15 is a graph of the crystallization level as a function of the addition amount and blending ratio of different types of compatibilizers. The crystallization level ($X_{c}$) of the resin was calculated using the formula below. It was confirmed that the high-hardness resin had a significant level of crystallinity, and the crystallinity remained relatively consistent even as the recycled TPEE resin was incorporated.

$$X_{c}=\frac{∆H_{f}}{∆H_{f}^{{}^{\circ}}w}\times 100$$

In our facility, because the fiber spinning process could only be carried out within the crystallization kinetics range of 45 to 60, blends of RT2, RT4, RT6, RT7, RT8, and RT9, with $X_{c}$ values ranging between 45 and 60, were promising candidates for fiber spinning. However, because the $X_{c}$ value serves as a reference value of the crystallization level for fiber spinning within our research group, careful examinations with other parameters are necessary in order to identify the most suitable materials for fiber spinning. *w* is the weight percentage of the polymer.

**Figure 16:**
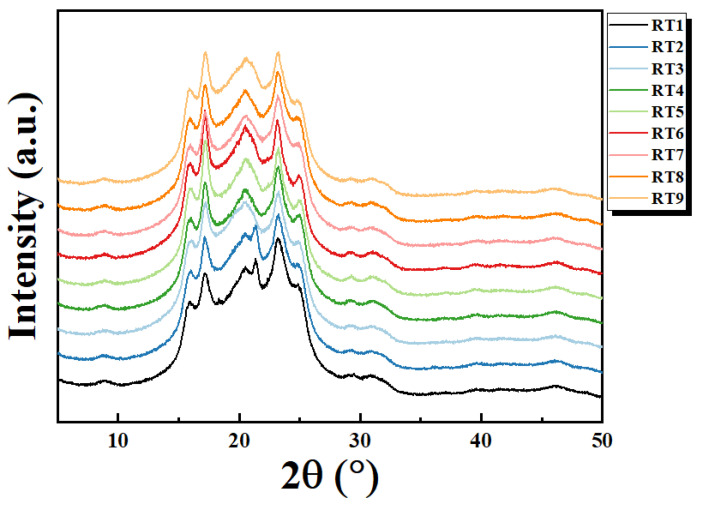
XRD measurement results according to the compatibilizer type and mixing ratio change.

As shown in Figure 16, XRD was performed via WAXD analysis (λ = 0.154 nm). TPEE showed a very weak diffraction peak at about 2θ = 15.9° regardless of the type of hardness (high or low). Additional diffraction peaks were also observed at 2θ = 16.4°, 17.9°, 21.3°, and 22.5°, rising from the PBT segment responsible for crystallinity in TPEE. In particular, the diffraction peak appeared clearly, indicating that the content of crystalline PBT was high. It was confirmed that even when the compatibilizer was mixed, there were no significant changes in peak movement or intensity. Accordingly, it was found that the structure of the material itself did not change even if the compatibilizer was added. The absence of such a structural change was the reason why the pyrolysis initiation temperature and melting point were similar regardless of the type or amount of compatibilizer added.

## 4. Conclusions

In this study, the following conclusions were obtained as a result of evaluating thermal and mechanical performance by mixing TPEE recycled resin and additives in various ratios with high hardness TPEE raw material to prepare a composition for use in fiber spinning, etc.

1. It was confirmed that the addition of recycled resin to the high-hardness resin resulted in a reduction of hardness compared to the raw material. Furthermore, the reduction in hardness remained negligible even when the amount of recycled resin was increased from 5% to 20%.
2. The virgin TPEE resin did not significantly increase the melt viscosity index even as the temperature was raised from 220 °C to 240 °C. In contrast, the melt viscosity index increased significantly with the increase in temperature for the specimens containing 20% R-TPEE. This phenomenon is attributed to the increase in the MFI value due to the decrease in the molecular weight of the added R-TPEE, which is believed to potentially lead to poor workability due to excessive flowability during product molding.
3. During the MFI evaluation, it was determined that the Elvaloy series compatibilizer showed the most suitability for fiber spinning, and Fusabond and Lotader series compatibilizers can also be used for fiber spinning if the addition amount is controlled.
4. The glass transition temperature of the Lotader and Elvaloy series compatibilizers was insufficient even with the change in the amount. Considering their thermal properties, these two series of compatibilizers are suitable for radiation. As a result of the evaluation of the overall characteristics, it is concluded that the Elvaloy-based compatibilizer is optimal for fiber spinning a TPEE composition containing 20% of R-TPEE.

## Figures and Tables

**Table 1:** Blending ratio of thermoplastic polyether–ester elastomer composition with compatibilizer added.

Specimen Numbers		RT1	RT2	RT3	RT4	RT5	RT6	RT7	RT8	RT9
**Base polymer**	TPEE	100	100	100	100	100	100	100	100	100
	R-TPEE	20	20	20	20	20	20	20	20	20
**Initiator**	DCP (Dicumyl peroxide)	-	0.05	0.05	0.05	0.05	0.05	0.05	0.05	0.05
**Compatilbilizer**	Fusabond N525	-	10	20	-	-	-	-	-	-
	Lotader AX8900	-	-	-	10	20	-	-	-	-
	Elvaloy 3427AC	-	-	-	-	-	10	20	-	-
	Lotryl 30BA02	-	-	-	-	-	-	-	10	20

## Data Availability

Not applicable.
